# Controlled Release of D-Limonene from Biodegradable Films with Enzymatic Treatment

**DOI:** 10.3390/polym17162238

**Published:** 2025-08-17

**Authors:** Viktor Nakonechnyi, Viktoriia Havryliak, Vira Lubenets

**Affiliations:** Department of Technology of Biological Active Substances, Pharmacy and Biotechnology, Lviv Polytechnic National University, Bandera 12, 79013 Lviv, Ukraine; viktoria.v.havryliak@lpnu.ua (V.H.); vira.i.lubenets@lpnu.ua (V.L.)

**Keywords:** carriers, gelatin, protease, D-limonene, MOS sensor, controlled release, digital twin, VOC

## Abstract

The instability of many volatile organic compounds (VOCs) limits their usage in different fragrance carriers and products. In scratch-and-sniff applications, VOCs are bound so strongly that release cannot happen without an external trigger. On the other hand, other fixatives like cyclodextrins release unstable volatile molecules too rapidly. We engineered biodegradable gelatin films whose release profile can be tuned by glycerol plasticization and alkaline protease degradation. Digitalized VOC release profiles acquired with the described near-real-time analysis toolkit are digital twins that replicate the behavior of the evaluated films in silico. Seven formulations were cast from 10% gelatin containing D-limonene, glycerol (5%, 20%), protease-C 30 kU mL^−1^, and samples with additional water to establish a higher hydromodule for protease catalytic activity. Release profiles were monitored for nine days at 23 ± 2 °C in parallel by metal-oxide semiconductor (MOS) e-noses, gravimetric weight loss, and near-infrared measurements (NIR). These continuous measurements were cross-checked with gel electrophoresis, FTIR spectroscopy, hardness tests, and sensory intensity ratings. Results showed acceleration of VOC release by enzymatic treatment during the first days, as well as overall impact on the release profile. Differences in low and high glycerol films were observed, and principal component analysis of NIR spectra separated low and high glycerol groups, mirroring the MOS and FTIR data. Usability of MOS data was explored in comparison to more biased and subjective intensity results from sensory panel evaluation. Overall, the created toolkit showed good cross-checked results and enabled the possibility for close to real-time analysis for bio-based VOC carriers.

## 1. Introduction

Design of new functional nanomaterials is emerging as part of innovations in biopolymer science. Deep learning, large language models, and other big data learning concepts, which are now commonly mentioned under the artificial intelligence concept, improve our ability to target narrow application niches. Consequently, researchers need materials that can be tailored with precision rather than one that fits all solutions. In parallel, digital tools like smart labels and embedded sensors become common components of materials research and scaling, enabling real-time monitoring and feedback during synthesis, processing, and end-user testing [1,2,3].

Biodegradable films for sustained release help reduce the use of plastics and toxic additives in everyday products, contributing directly to sustainability goals [4]. New understandings and advances in VOC release dynamics decrease the usage of chemically synthesized materials and shift it to natural alternatives. Research into novel controlled release systems remains highly relevant, and applying near-real-time advanced data processing approaches can both review current achievements and improve existing solutions [5,6].

Unstable volatile molecules such as D-limonene or cis-3-hexen-1-ol and differently perceived eugenol pose challenges for controlled release from biodegradable materials due to high volatility and complexity of perception, odor threshold. The majority of Flavor and Fragrance industry companies have investigated systems of controlled release for a long period, and a large number of new patents in this field continue to appear in recent years, but mostly driven by incumbents of the market, which highlight interest in this domain and the importance of this exploration [7].

Biodegradability, tunable mechanical properties, and availability make gelatin one of the biopolymers that is interesting for exploration as a VOC carrier component. Gelatin is typically produced from by-products such as skin or bones and obtained by hydrolyzing collagen. Variations in bloom strength are common from 50 g to over 300 g bloom and corresponding to molecular weights 40–300 kDa, which allows for tuning of gelation temperature, mesh size, and, as a result, can influence release profiles [8].

New materials must have a certain level of flexibility to be used as a source for new product developments. Glycerol is one of the low-cost, edible, and commonly used plasticizers. Its hydrophilic nature enables it to spread among gelatin macromolecules and expand intermolecular spacing as a result, enhancing film flexibility. Three hydroxyl groups create physicochemical properties such as high viscosity, water miscibility, and plasticizing ability [9,10].

D-limonene is one of the common components of essential oils, which are recently gaining more interest due to their naturalness and sustainability. Because it is a small molecule and prone to oxidation, it is highly unstable and difficult to maintain over extended periods in various delivery matrices [11].

In order to have more functional components in biodegradable materials, enzymatic treatment can be used. Protease-C is a broad-spectrum enzyme that hydrolyzes the peptide bonds within protein matrices. In gelatin films, it cleaves long collagen-derived polypeptide chains into shorter fragments and effectively increases network porosity as well as facilitating the sustained release of encapsulated actives [12,13].

In this study, we evaluated release profiles of gelatin/glycerol films functionalized with D-limonene and enhanced with protease-C. The detailed characterization of the glycerol and protease impact on the resulting active films is provided. Main innovative aspects in this study reported here includes applying near-real-time or continuous daily based measurements for prepared gelatin/glycerol/protease/limonene films using panel of metal oxide semiconductor sensors (MOS) and near infrared spectra sensors (NIR), in depth data processing of Fourier transform infrared (FTIR) spectroscopy, effect of protease as an accelerator for volatile organic compounds diffusion from gelatin matrices and films preparation.

## 2. Materials and Methods

### 2.1. Materials

High molecular gelatin type A, CAS 9000-70-8, 270 bloom, was sourced from TROBAS GELATINE BV (Dongen, The Netherlands). Glycerol with CAS number 56-81-5 and 99.5% purity was purchased from SferaSim (Lviv, Ukraine). Alkaline protease-C with activity > 30 K (#01-25) was purchased from Enzim Biotech (Ladyzhyn, Ukraine), and D-limonene with CAS number 5989-27-5 was obtained from UkrAroma (Dnipro, Ukraine). In experiments, we also used such reagents: Tris, acrylamide, N,N′-methylene-bis-acrylamide, persulfate ammonium, 2-mercaptoethanol, and glycerol were from ACROS Organics (Geel, Belgium); sodium dodecyl sulfate—Applied Chem (Lviv, Ukraine). Coomassie Brilliant Blue R-250, bromophenol blue, glacial acetic acid, and hydrochloric acid were purchased from SIMKO Ltd. (Lviv, Ukraine). All chemicals and reagents used were of analytical grade.

### 2.2. Film Development

An IKA Magnetic Stirrer RO 15 (IKA-Werke GmbH & Co, Staufen im Breisgau, Germany) with a speed range of 0–1200 rpm was employed for the film’s development. To obtain a 10% (*w*/*v*) gelatin solution, gelatin was first swollen in distilled water for 10 min, then dissolved in a 55 °C water bath for a further 10 min. Film casting emulsions were produced as follows. The 30 mL of the hot 10% gelatin solution and 10 mL of D-limonene were stirred at 480 rpm for 10 min as the baseline formulation, L@Gel. Glycerol was then introduced at either 5% or 20% of the gelatin dry mass (labelled /0.5Gl and /2Gl, respectively) and mixed for an additional 5 min. For enzyme-containing films, 0.5 mL of protease-C (3 K U mL^−1^) was added, and the dispersion was stirred for a further 5 min (/PT). For the protease-treated samples, an extra 10 mL of the distilled water was added and stirred for 5 min to raise the hydromodule and enhance catalytic activity; these samples are labeled with /PTxW. The final emulsions were poured onto poly(methyl methacrylate) plates and levelled to a wet height of 1 mm. Drying was carried out at 22 °C and 55% relative humidity for 25–30 h, and emulsions had a moderately acidic pH of 4–5. Table 1 presents material names and components.

### 2.3. Physicochemical Characterization, Mechanical Properties, Gravimetric Analysis, and Sensory Evaluation of Films

#### 2.3.1. Fourier Transform Infrared Spectroscopy

Prepared films were analyzed using Fourier transform infrared spectroscopy (FTIR) by PerkinElmer Spectrum Two (710 Bridgeport Avenue, Shelton, CT 06484-4794, USA). Raw data of measurements extracted in the format of *.asc files and analyzed.

Each film size 25 × 60 mm was pressed onto the crystal with the built-in pressure arm for uniform contact. A background spectrum of the clean crystal was recorded before every sample. Spectra were collected from 4000 to 400 cm^−1^ using automatic gain optimization “AutoGain” at a nominal resolution of 4 cm^−1^ with averaging 32 interferograms per measurement. All spectra were obtained at 20 ± 1 °C.

For two film groups with and without protease, the mean spectrum was calculated. The difference spectrum was then computed, and regions corresponding to the top 5% of absolute differences were highlighted as wavenumbers where changes most strongly affect the FTIR profile. FTIR data files and algorithms added to the Supplementary Materials.

#### 2.3.2. Gravimetric Analysis and Optical Images

Gravimetric analysis of weight loss for films was conducted using an analytical grade weigher RADWAG AS220 R2 (Radwag, Radom, Poland), and recording mass to three decimal places. The test samples were measured while placed on Petri dishes. The measurement data are provided in the Supplementary Materials.

Optical visuals were created with microscopy images of film surfaces using a microscope, MICROmed XS-5520 LED (Kyiv, Ukraine).

#### 2.3.3. Metal-Oxide Sensors

Release kinetics were analyzed using seven portable MOS sensor arrays in parallel, ENS160 (ScioSense, Eindhoven, The Netherlands), known as an e-nose. Each sensor has four heated micro hotplates and integrated baseline correction software, as well as on-chip humidity and temperature compensation. The sensor has a ppb-level total VOC value after 3 min of warm-up. The output range of the sensor is 0–65 K ppb with a ± (0.12 × reading) 1 σ uncertainty, and sensing plates are mainly based on materials SnO_2_, ZnO, CuO, and TiO_2_.

Sensors have been powered for 24 h at room temperature before measurements to stabilize the baseline resistance. Then sensors were subsequently verified for consistency, for one minute with a one-second measurement interval; their readings differed by no more than ±10 ppb. Measurements were conducted in a room 40 m^2^ with 7 sensors running measurements in parallel for 7 different film samples of size 25 × 60 mm surface area in containers 40 × 30 × 25 cm with a distance between sensor and sample of 27 cm, indoor temperature 23 ± 2 °C. Calibration for D-limonene was performed by placing 0.1 mL of D-limonene on a Petri dish and measuring it under the same sensor setup and environmental conditions described above. In order to analyze each second-by-second data from sensors and normalize the daily temperature peaks impact, total volatile organic compounds (TVOC) readings were normalized by subtracting the 10th percentile value within a 5-s window and then averaging the adjusted values for each hour.

#### 2.3.4. Near-Infrared (NIR) Spectroscopy

NIR spectra have been evaluated once per day using an AS7263 NIR/VIS spectral sensor, which detects wavelengths below the visible range with 6 near-IR channels: 610 nm, 680 nm, 730 nm, 760 nm, 810 nm, and 860 nm. Analysis conducted on all channels, but the 810–860 ranges are most interesting due to possible indirect D-limonene validation. Before every measurement session, the sensor was warmed for 3 min, and the dark stage was recorded with the illumination LEDs off and subtracted. Film test samples 25 × 60 mm were placed on a black stage and evaluated at a fixed 15 mm working distance with the sensor’s default LED light. The measurement data are provided in the Supplementary Materials.

#### 2.3.5. Sensory Evaluation

Sensory evaluation was conducted by 6 panelists with an assessment of the intensity scale between 0–10 to understand the trend of odor intensity changes over time, and overall human feedback and notes. To train panelists for intensity values, jojoba oil has been used to mix with D-limonene: 0—odor free, 10—1 mL of D-limonene for 1 g of oil, and intermediate 0.2, 0.4, 0.6, 0.8 mL per 1 g. After training, panelists used to evaluate film samples of the 25 × 60 mm surface area in a room 50 m^2^ and temperature 23 ± 2 °C, placed on Petri dishes.

#### 2.3.6. Hardness Tests

Hardness was measured by the Shore A durometer hardness test method, using a durometer PCE-DD-A Hardness Tester with a 35° truncated cone indenter, a measurement scale of 0–100, a resolution is 0.5, and ±2 variation. The durometer method uses a spring-loaded truncated cone indenter that is pressed into the surface with a defined force, and the depth of penetration is converted into a hardness value on the Shore A scale (ShA).

Samples were prepared by cutting the film into slices and stacking them to achieve a total thickness of 6 mm for further measurement with a durometer. Multiple measurements are taken at different locations and averaged for analysis.

#### 2.3.7. Gel-Electrophoresis

Films were analyzed by electrophoresis in a 12.5% polyacrylamide gel under denaturation conditions with sodium dodecyl sulfate (SDS-PAGE) in the Laemmli buffer system. The samples of films were dissolved in boiled distilled water. After cooling, the solutions were mixed with a loading buffer (0.5 M Tris-HCl (pH 6.8), glycerol (20% *v*/*v*), SDS (5% *v*/*v*), bromophenol (0.01%), β-mercaptoethanol (ME, 5% *v*/*v*) at a ratio of 1:1 (*v*/*v*). Then the mixtures were incubated at 90 °C for 5 min and loaded onto the gel. After the separation of proteins, the gel was stained with Coomassie R-250 for 1 h and then washed with a solution of 7% acetic acid.

### 2.4. Statistical Analysis

Hardness (shore A scale), % weight loss, pH, sensory analysis (intensity of scent by scale 0–10 points), means values, as well as their standard deviation, were calculated. These properties were also subjected to further statistical analysis using the ANOVA with the Tukey HSD method to investigate statistical differences between measurement mean values. Assuming a significance level of *p* < 0.05, all measurements were conducted using *n* = 3 samples of each L@Gel, L@Gel/0.5Gl, L@Gel/0.5Gl/PT, L@Gel/0.5Gl/PTxW, L@Gel/2Gl, L@Gel/2Gl/PT, L@Gel/2Gl/PTxW film. Statistical analysis and PCA were performed using the Scikit-learn software library (version 1.2.0).

## 3. Results

### 3.1. Film Surfaces and Impact of Preparation Method

Stirring speed sets the microstructure of the gelatin/D-limonene emulsion and controls droplet size. Low speed, less than 200 rpm, and less than 2 min, leaves millimeter-scale droplets that rise during drying. High speed 6–10 K rpm for 5–10 min breaks the oil into smaller 1–5 µm droplets. Larger speeds up to 15 K rpm and more than 15 min might cause excess energy with possible emulsion overheating, and droplet flocculation during cooling produces aggregates that dry into pits [14,15]. Stirring time also impacts the viscosity of the solution on the cast. Thinner films are more transparent.

**Figure 1:**
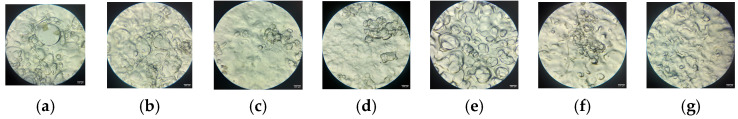
Optical images of films, scale bar: 100 µm, (**a**) L@Gel, (**b**) L@Gel/0.5Gl, (**c**) L@Gel/0.5Gl/PT, (**d**) L@Gel/0.5Gl/PTxW, (**e**) L@Gel/2Gl, (**f**) L@Gel/2Gl/PT, (**g**) L@Gel/2Gl/PTxW. Samples (**c**,**d**,**f**,**g**) containing protease-C might exhibit a more pronounced yellow color due to the enzyme’s impact.

Glycerol acts primarily as a plasticizer and emulsifier in gelatin. Its addition increases viscosity, stabilizing the emulsion and preventing rapid droplet coalescence. The increased viscosity from glycerol slows down droplet migration during drying, resulting in a more homogeneous film and smaller, uniform droplets. Films incorporated with glycerol typically exhibit a smoother surface. Figure 1 shows the optical images of prepared films with a stirring speed of 480 rpm for 5 min. Samples L@Gel, L@Gel/0.5Gl, and L@Gel/2Gl reveal larger surface droplets compared to films treated with protease. Few studies also mentioned that in protease-treated films, the partial hydrolysis of gelatin lowers its viscosity and supplies small amphiphilic peptides that migrate to the oil/water interface, producing a finer internal emulsion and leaving the surface largely free of large droplets [16,17,18].

### 3.2. D-Limonene Stability and Rate of Evaporation, Protease-C Tests

**Figure 2:**
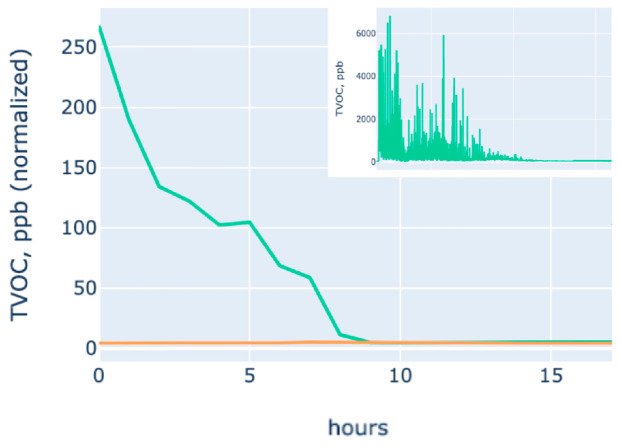
D-limonene diffusion plot (in ppb) measured with the MOS sensor by hours, values are normalized by reducing the base by the 10th percentile on a 5 s time window, and after being averaged per hour (in the corner chart are original raw values from the sensor). The bottom chart’s yellow line shows a non-loaded sensor.

Volatility of D-limonene is high, 0.1 mL under 23 °C evaporates in around 6–8 h, approximately as shown in Figure 2 with MOS sensor raw and normalized values.

D-limonene is a purely hydrocarbon monoterpene with two internal C=C double bonds and no hetero-atoms, small as 136 g mol^−1^. Volatility boosting happens over weak cohesive forces with only C-H bonds and no sites for hydrogen-bonding or strong dipole interactions, compact and branched geometry, and ten carbons keep the vapour pressure high (2 mm Hg at 20 °C, 270 Pa). Henry constant (H = 3.8 × 10^−4^ mol m^−3^ Pa^−1^) shows that at 25 °C, limonene prefers the gas phase by a factor of 35,000 over water, so once it reaches the film surface, it moves into air almost exclusively. Aerial autoxidation of D-limonene happens via radical abstraction at C-6, C-7, forming peroxy radicals that convert to the limonene-1,2-oxide, carveol, and carvone, while trace acids catalyze isomerization to terpinenes [19].

Oxidation by OH radicals is slower because typical indoor [OH] is only 10^5^–10^6^ molecules cm^−3^ [20]. With usual residential and small-lab room ventilation at 0.5–2 air changes per hour, VOC will be removed faster than can be achieved by gas phase oxidation with O_3_ and OH.

Some studies mention that protein-rich gluten films and gelatin/PLA composites improve VOC retention by keeping >70% of the terpene unoxidized after 21 days at 25 °C by scavenging radicals and limiting O_2_ ingress [21].

From a bioengineering perspective, prolonging D-limonene functionality hinges on minimizing its initiated oxidation by oxygen/moisture barriers, scavengers, or inclusion hosts and tuning film diffusivity so that outward flux remains above odor threshold around 20–50 ppb, also below the oxidation rate constant by decoupling aroma release from chemical degradation.

Protease C-1 is a fungal alkaline serine protease obtained from *Acremonium chrysogenum*. Its activity peaks at pH 8.0–10.5 and 50–60 °C, and it remains catalytically competent over pH 5.5–11.5 and 30–70 °C. Catalysis follows the subtilisin-like mechanism in which Ser195 nucleophilically attacks peptide carbonyls to form an acyl enzyme that is hydrolyzed by water; however also initiates autoproteolysis, making water activity the critical factor between stability and self-digestion [22].

Immobilization strategies moderate this behavior, for example, adsorption/cross-linking on chitosan films preserves 94% of the initial activity and throttles enzyme diffusion, showing quasi-zero-order erosion of gelatin over 10–14 days. Alkaline protease bound to zeolite silica retains 74% activity after 16 days versus 50% for the free enzyme [23,24].

**Figure 3:**
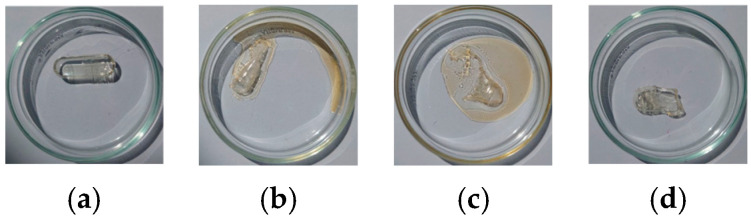
Impact of protease-C load into soft gelatin capsule (samples (**b**,**c**): 0.3 mL protease-C 3 K U mL^−1^:0.5 mL D-limonene and 0.5 mL protease-C 3 K U mL^−1^:0.3 mL D-limonene) versus solo D-limonene in gelatin capsule (**a**) and solo water in gelatin capsule (**d**).

To verify that gelatin can efficiently encapsulate D-limonene and retain it over time, also to check the impact of protease on the gelatin matrix, we performed a high-level experiment using soft gelatin capsules. Figure 3 shows the impacts of gelatin capsule loadings. Sample (a) keeps loaded D-limonene inside the capsule for weeks to months, while adding the enzyme solution (b,c) releases D-limonene in a few hours by degrading the capsule. This proves that gelatin can retain volatile molecules for a long time, as well as that protease can enhance release. Sample (d) control one with water loaded into a gelatin capsule; in a few hours, water is released, and the capsule shape has changed.

### 3.3. Physicochemical Analysis of Bio-Films

#### 3.3.1. FTIR Analysis

Spectra analysis was conducted in four parts: first, the regions of interest were found based on film components, then glycerol bands were checked, next, D-limonene content was evaluated, and finally, peak analysis was modeled to examine protease activity.

**Figure 4:**
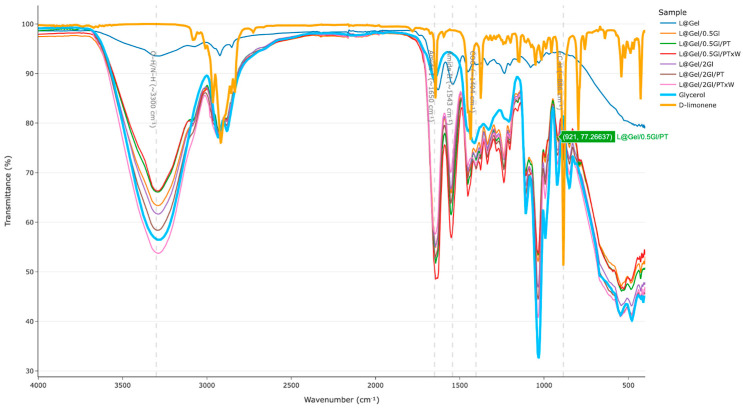
FTIR spectra for films and separately glycerol and D-limonene, taken on the 3rd day after film casting.

In Figure 4 broad FTIR spectra are shown with highlighted regions of interest: broad absorption region around 3300–3280 cm^−1^ from O-H/N-H stretching amide A of peptide bonds and bound water [25]; amide I/II peaks at 1651 cm^−1^ and 1543 cm^−1^ [26]; strong C-O stretching vibrations in the fingerprint region related to glycerol as literature reports an intensified band near 1030–1040 cm^−1^ in glycerol plasticized gelatin films [25]; identification regions for D-limonene are 870–890 cm^−1^, 1643 cm^−1^, 2832 cm^−1^, 2913 cm^−1^ and few others but these most common [27]; enzymatic hydrolysis may attribute to the accumulation of new COO^−^ groups 1390–1405 cm^−1^ [28] or an N-H bending of -NH_3_^+^ can appear around 1510 cm^−1^ [29].

**Figure 5:**
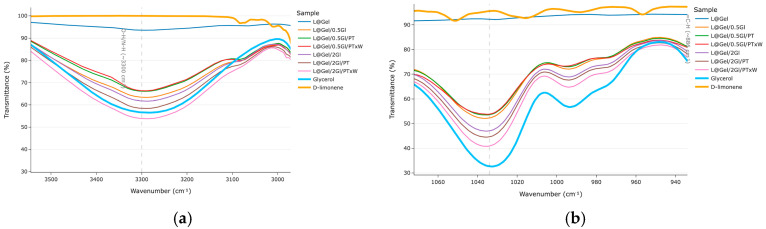
FTIR wavelength windows based on identified regions of impact: (**a**) range O-H/N-H, 3300–3280 cm^−1^ (**b**) glycerol, stretch at 1030 cm^−1^. Glycerol and D-limonene are added for reference and are raw materials.

Peaks related to glycerol validation are presented in Figure 5. For films without enzyme, the C-O stretch at 1030 cm^−1^ decreased from 52% T (5 wt% glycerol) to 46% T (20 wt% glycerol), corresponding to an 11% intensity difference; from protease-treated films L@Gel/2Gl/PTxW showing the largest peak there 40% T. Similar behavior observed at 3210–3450 cm^−1^, L@Gel/2Gl/PTxW showing the largest peak 53% T.

**Figure 6:**
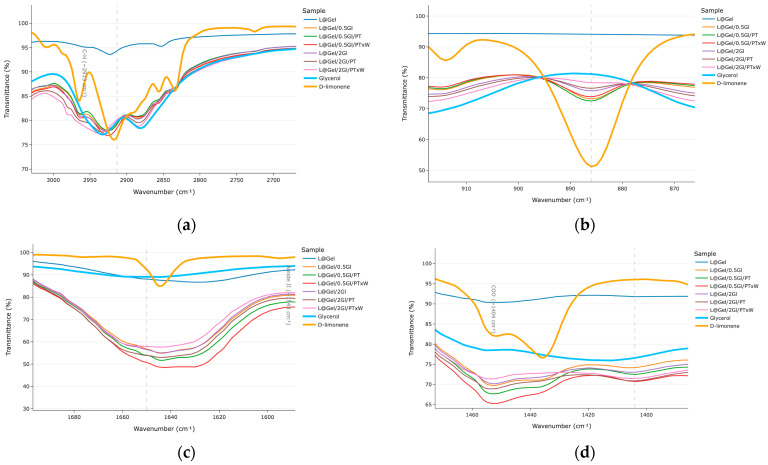
FTIR wavelength windows based on identified regions of impact: (**a**) C-H 2913 cm^−1^, (**b**) =C-H at 887 cm^−1^, (**c**) 1643 cm^−1^ for D-limonene identification, (**d**) 1404 cm^−1^ COO^−^ range. Glycerol and D-limonene are added for reference and are raw materials.

In Figure 6, the presented peaks contribute to D-limonene evaluation. At 2913 cm^−1^ all films show similar transmittance (79–80% T), with slightly higher peak L@Gel/0.5Gl/PTxW of 78% T. Marker region around 887 cm^−1^ where reference D-limonene has 51% T peak, the group of films with lower (5% glycerol) having peaks roughly 6% higher than those with 20 wt% glycerol which is indicating terpene findings in the low-plasticizer matrix. At 1644 cm^−1,^ films L@Gel/0.5Gl/PTxW and L@Gel/0.5Gl/PT have values 48% T, 51% T, respectively.

In the COO^−^ stretching region in Figure 6d, the samples L@Gel/0.5Gl/PTxW, L@Gel/2Gl/PTxW, and L@Gel/2Gl/PT all display identical peak intensities of 71% T, whereas the corresponding band for every formulation lies within a narrow 70–74% T window.

**Figure 7:**
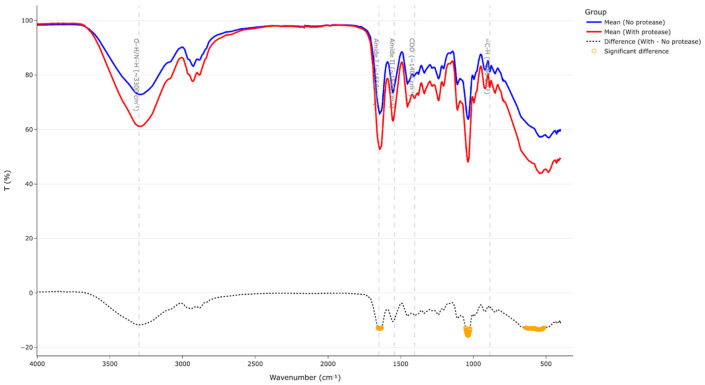
FTIR peak aggregated data for films with protease-C and without, the grey dashed line highlights spectra differences.

To analyze the impact of protease-C based on multiple bands of FTIR data (not easy to visually observe without data preparation), we performed grouping for samples with protease-C and without, and highlighted differences (algorithm added in Supplementary Materials). In Figure 7 bottom plot line highlights in yellow the differences between films with added protease-C and without.

D-limonene is not well solubilized in glycerol and gelatin film matrices; some of it might migrate to the surface during drying because the drying front can carry hydrophobic droplets outward. Researchers have noted essential oils can migrate to one surface during film drying, depending on drying rate and emulsion stability [30]. A low glycerol film likely dries faster with less plasticizer to hold water, potentially concentrating limonene near the drying surface. It is also possible that the high glycerol film is more moisturized when pressed and might dilute primarily water/glycerol to the surface. For the PerkinElmer Spectrum 2 fitted with its internal Universal ATR, the infrared wave possibly penetrates the top 0.5–2 µm of the sample surface layer.

#### 3.3.2. Gel-Electrophoresis

Literature data indicate that high bloom gelatin (250 g bloom) is dominated by intact α-chains (100–120 kDa) and β-dimers (around 200 kDa). When exposed to protease C, the bulky α- and β- bands are reduced within 30–60 min and replaced by smaller 20–40 kDa bands as multiple cleavages occur after Leu-, Phe-, and Tyr- [31].

**Figure 8:**
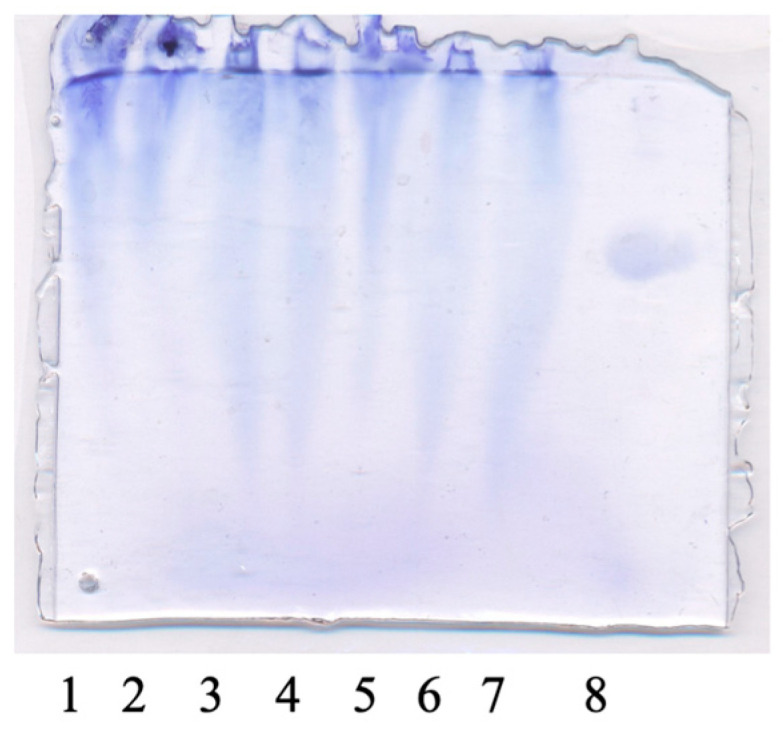
Gel-electrophoresis of films in order from left to right (lanes 1–7): L@Gel, L@Gel/0.5Gl, L@Gel/0.5Gl/PT, L@Gel/0.5Gl/PTxW, L@Gel/2Gl, L@Gel/2Gl/PT, L@Gel/2Gl/PTxW, lane 8—albumin with molecular weight 66.5–67 kDa.

Figure 8 shows the electrophoretic profiles of the obtained films. These patterns showed fuzzy bands, but their intensity varied. The presence of an intense band in the upper part of the gel, corresponding to a molecular weight above 67 kDa, may indicate the presence of α-chains. Some authors reported that the electrophoretic bands with an MW of ~ 116 kDa can be described as α-peptide chains ~ 100 kDa of collagen [32]. The bands in the low-molecular-weight protein region are more pronounced in lanes 3 and 4, as well as lanes 6 and 7. It should be noted that these lanes correspond to samples treated with protease C.

### 3.4. D-Limonene Release from Bio-Films

#### 3.4.1. Release Kinetics

MOS sensors were employed to analyze the diffusion rate of D-limonene from film matrices. All film samples were measured in parallel to minimize the influence of environmental factors such as daily fluctuations in temperature and humidity. While some environmental variations still exist, their effects are relative consistent across the film samples. For experiments, increasing the number of available sensors may eventually prove to be a more effective strategy than relying on complex calibration entropy. Alignment between human scent perception and TVOC sensor readings was consistently observed within 24 h for a sample of 0.1 mL of D-limonene.

**Figure 9:**
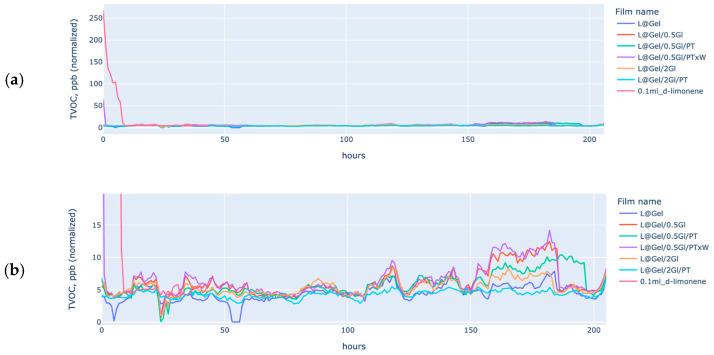
Plot (**a**) shows the full range of overall normalized TVOC, ppb (normalization approach described with Figure 2), Plot (**b**) same films, data is zoomed in to be in the range of 0–15 normalized TVOC ppb values.

Plot B on Figure 9, samples L@Gel/0.5GI/PTxW, L@Gel/0.5Gl, and L@Gel/0.5Gl/PT are the top three by normalized ppb values.

Observed larger burst releases for L@Gel/0.5Gl/PTxW with protease and additional water, which are also aligned with weight loss, which is the indirect proof of TVOC measurements. After 100 h, some samples start to be intersected without showing larger or more significant differences, but some still show lower grades of release. Area under the curve (AUC) for 0.1 mL D-limonene in Figure 7 plot shows VOC evaporation during 6–8 h. By computing the proportion of initial D-limonene which is used for film creation (10 mL) and % of weight loss during film drying after the first two days after film casting, which is around 30%, then we can assume that a 1 g film sample may have a payload of D-limonene to be around 0.3 mL. By approximating that 0.1 mL with low-to-moderate release can evaporate during 30 h, 3× times the payload may achieve recognizable scent release up to 90 h, which is around 4 days.

#### 3.4.2. Daily NIR Analysis

The diagnostic window, 800–900 nm range accessible by the NIR sensor used in this study. Wavelength 810 nm is associated with C-H_2_ methylene groups and aromatic C-H, 860 nm is typically linked to C-H_3_ methyl groups and aliphatic C-H chains; both of them should be able to detect hydrocarbons like terpenes. Few studies attributed 830 nm to C-H third overtones in lipid-rich or terpene-bearing systems, used chemometric analysis for quantitative D-limonene content analysis in spray dried powders, and described peaks at 830–870 nm attributed to C-H_3_/C-H_2_ of D-limonene [33].

**Figure 10:**
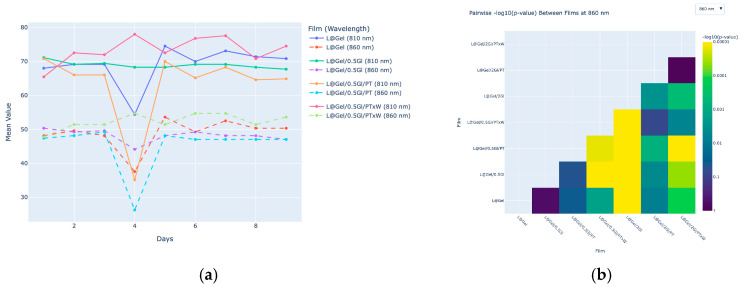
NIR charts: (**a**) daily values for NIR measurements of 810 and 860 nm over 9 days for films L@Gel, L@Gel/0.5Gl, L@Gel/0.5Gl/PT, L@Gel/0.5Gl/PTxW put on the same chart. (**b**) pairwise significance of difference between films on 810 nm (smaller value, larger difference).

Since possible overlaps may happen for compounds like D-limonene and glycerol due to similar groups, for example, sharp C-H_3_ may overlap glycerol’s broad C-H band, Figure 10 presents a comparison of daily measurements between films with the same or relatively small differences in glycerol amount. Observed that L@Gel/0.5Gl/PTxW shows constantly higher values over days for 810, 860 nm wavelengths. Additionally, no significant trend for growth or decline can be defined based on 9 days of measurements.

**Figure 11:**
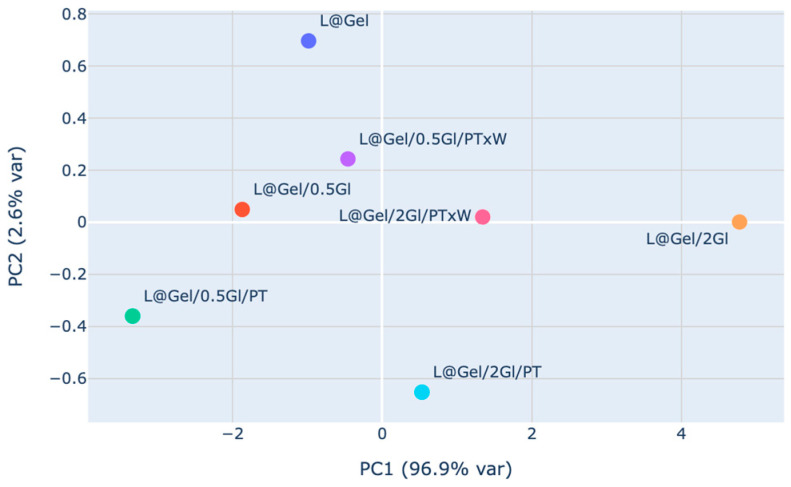
PCA analysis of NIR data for samples: L@Gel, L@Gel/0.5Gl, L@Gel/0.5Gl/PT, L@Gel/0.5Gl/PTxW, L@Gel/2Gl, L@Gel/2Gl/PT, L@Gel/2Gl/PTxW.

Principal component analysis (PCA) was performed on standardized six waves of NIR spectral data to reduce dimensionality and visualize the main sources of variance among samples. Features were normalized using a standard scaler for comparability across wavelengths. The first two principal components accounting for the largest variance in the dataset were extracted in Figure 11.

The first principal component, PC1, accounts for most of the variance, 96.9%, and the second component, PC2, explains a much smaller proportion, 2.6%. Most samples are separated along the PC1 axis, indicating that the primary differences in NIR spectral profiles among the films are captured by this component. Samples L@Gel/2Gl/PT and L@Gel/0.5Gl/PT are positioned at opposite extremes of PC1, showing substantial spectral differences between these formulations. Samples L@Gel/0.5Gl/PTxW and L@Gel/2Gl/PTxW cluster closer together, indicating more similar NIR characteristics. The relatively small spread along PC2 suggests that secondary sources of variation are minor compared to those captured by PC1.

#### 3.4.3. Gravimetric Analysis for Film Weight Loss

Gravimetric analysis of weight loss for each film has been performed. As the main base component of all films is gelatin, it shows a tendency to have some minor weight fluctuations by gaining and losing weight, which responds to environmental indoor conditions like humidity changes from day to day. These fluctuations are minor and do not significantly impact the final end-of-period weight loss between films. For films with larger gelatin context (L@Gel/2Gl, L@Gel/2Gl/PT, L@Gel/2Gl/PTxW), weight variability is larger because of the viscosity of the solution at the time of casting. Glycerol percentage impacts moisture uptake and can reach around 15–20% with increased relative humidity (RH) to 57% as mentioned in some studies [34,35,36]. Table 2 shows a comparison of films weight loss in % over nine days.

Films with additional water and protease-C showing larger % weight loss during first 2–3 days, however additional water is only 20% of L@Gel/0.5Gl/PTxW compared to L@Gel/0.5Gl/PT but overall weight loss in measured days close to approximately 50%, so it can be indirect proof that more D-limonene used to be released. This is also aligned with MOS charts, where an initial larger burst-release for L@Gel/0.5Gl/PTxW.

#### 3.4.4. Sensory Analysis

VOC release perception from biodegradable films is a function of many factors, such as film components and method of preparation, air flows, personal attention, and mood at the time of measurement, longevity of validation, all of which add extra subjectivity for sensory data analysis. Studies mention that sensory panelists are highly variable and very prone to bias, as well as that “simulations of sniffing versus quiet breathing demonstrate that sniffing delivers about 2.5 times more air to the olfactory recess and results in 2.5–3 times more uptake of odorants per unit time” [37,38]. Often, for VOC diffusion, observed patterns are “burst then plateau” release controlled by plasticiser level, film porosity, and droplet binding.

**Figure 12:**
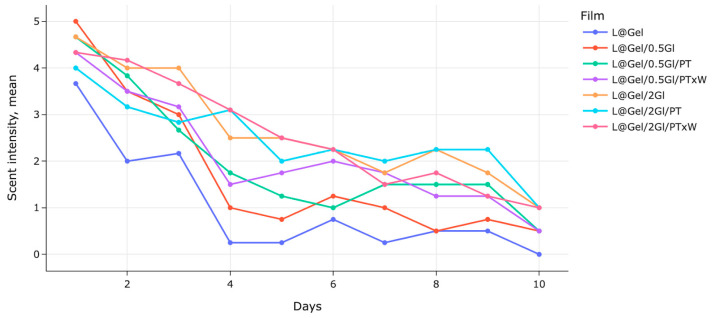
Sensory panel mean intensity values of 3 batches of measurement over 10 days with indoor conditions between 20–25 °C: L@Gel, L@Gel/0.5Gl, L@Gel/0.5Gl/PT, L@Gel/0.5Gl/PTxW, L@Gel/2Gl, L@Gel/2Gl/PT, L@Gel/2Gl/PTxW.

Figure 12 shows mean intensity values over 3 batches of measurements with daily values, with an intensity range between 0 to 10. Sensory panel results show that VOC release for low and high glycerol content has notable differences in the perception of how it releases scent. At the same time, variance in sensory reports is high between all film samples, most of which cannot be marked as statistically different with the default *p* < 0.05. Panelists mentioned that films L@Gel/0.5Gl, L@Gel/0.5Gl/PT and L@Gel/0.5Gl/PTxW have more notable scent in 9 days compared to L@Gel/2Gl, L@Gel/2Gl/PT, L@Gel/2Gl/PTxW with high glycerol content, however observation that these films are also more flexible and release more scent when they are touched by hands due to mechanical changes.

From studies, less than 10% wt plasticised films may trap aroma for longer, while at higher glycerol contents > 20 wt% gelatin matrix becomes more flexible and less densely packed, there is more free volume in the film matrix, and VOC should evaporate quicker. Glycerol’s hydrophilicity makes films more attractive to water; higher glycerol films tend to absorb more moisture from the environment, becoming thicker and more hydrated [10,39,40]. Research on chitosan films with aroma compounds found that “aroma retention during film drying was directly related to the water content” [41]. Glycerol slows the drying rate because it binds water and makes the film-forming solution more viscous, which might impact less D-limonene flashes off immediately, and this might make an impact in a way that glycerol shifts the timing of D-limonene release. Moreover, as glycerol is hydrophilic, it should not solubilize D-limonene greatly.

### 3.5. Mechanical Parameters

Mechanical properties were evaluated using a durometer hardness test. Hardness values for the seven films in Table 3 show a clear softening gradient from the gelatin/D-limonene control 83.3 ShA, which already contains 25 wt% of D-limonene droplets and retains high hardness, to the high glycerol with protease and extra water formulation 41 ShA. Raising glycerol to 20 wt% cuts hardness by 40 ShA from 83 to 50, a 40–50% loss that aligns with other study data showing linear drops in tensile strength and Young’s modulus when glycerol reaches 20–30% of gelatin solids [42]. Researchers observed that fish skin and pepsin-modified gelatins show 25–40% lower gel strength and hardness compared with untreated controls [43], which is closely aligned with data for our films.

## 4. Discussion

In this work, we explored how protease treatment and varying glycerol concentrations remodeled gelatin film structure, altered mechanical properties, and modulated the release kinetics of the hydrophobic molecule D-limonene.

Spectroscopically, all samples showed the common gelatin amide I/II bands at 1651 cm^−1^, 1543 cm^−1^ together with the 3300 cm^−1^ OH/NH peaks. Increasing glycerol from 5 wt% to 20 wt% amplified the C-O stretch around 1032 cm^−1^, showing successful plasticization. D-limonene fingerprints at 887, 1643, and 2913 cm^−1^ were detectable and showed 6% larger peaks for films with 5% glycerol compared to 20% glycerol films, which is also a discussion of measurement approach in terms of how much D-limonene on the surface versus full film content impacts FTIR results. Film with 5% glycerol and protease with extra water showing an increase in D-limonene peak on FTIR compared to others in the same glycerol amount group, which also aligns with MOS data measurements.

VOC release profiles extracted from the MOS data follow the classic burst and plateau shape, where L@Gel/0.5Gl/PTxW released the highest TVOC signal within the first 48 h and a less significant overall hourly release rate during all periods. It outperformed their 20 wt% glycerol analogues as well as non-protease films and pointing out that a looser but more hydrated matrix may show slower diffusion, or more D-limonene released during film casting, which can also be seen on the 887 cm^−1^ FTIR band. Weight loss data show quantitatively that films that lost more than 8% mass in nine days were exactly those exhibiting the steepest initial VOC slopes.

Despite the NIR measurements, absolute changes at 810 nm and 860 nm were modest; PCA reduced 96.9% of spectral variance into PC1 and cleanly separated low from high glycerol films, which aligned with FTIR data.

Mechanically softening gradient identified by raising glycerol to 20 wt% or introducing protease lowered Shore A hardness by up to 50%. From the analyzed films, the softest film with 41 ShA exceeds the minimum required for handling as for example, for odor strip or inner sachet. The control L@Gel 83 ShA kept hardness but released the least D-limonene. Such data underscore the tradeoff between barrier integrity and diffusion, where designers should tune film components, as in our example, plasticiser and enzyme levels, not in isolation but as coupled levers that shift both mechanics and release kinetics.

Sensory evaluation of scent intensity is subjective and quite hard to measure, so probably in some cases more convenient to rely on weight loss and MOS data. Protease-treated films show smoother surfaces with fewer large droplets, which may point to denser microstructure. Stirring speed is an important parameter that impacts the initial oil droplets distribution and should be evaluated further. In-depth data of explored objects brings more insights and helps understand mechanics, while at the same time, it might be beneficial that a broader variety of film components may introduce larger variances for target functions.

## 5. Conclusions

In conclusion, gelatin films plasticised with 5 wt% glycerol and featured with protease-C plus 10 mL of water L@Gel/0.5Gl/PTxW delivered the highest initial D-limonene spike compared to the enzyme-free analogue and maintained acceptable flexibility of 52 ShA. Glycerol levels of 5% and 20% largely impact film properties and scent release behavior. A mix of near real-time techniques like MOS and NIR with gravimetric weight loss analysis, which shows alignment between results, presents a toolkit for dynamic, time-series evaluation of highly volatile molecules retention and release, like terpenes, as well as active biofilm behavior. From an application perspective, developed films and a near-real-time analysis approach may be further explored as a ground for a scalable scent delivery platform by designing and assembling multilayer film structures.

## Figures and Tables

**Table 1:** Film names and components.

Film Name	Gelatin	D-Limonene	Water	Glycerol	Protease-C	Extra Water
L@Gel	10 g	10 mL	10 mL	-	-	-
L@Gel/0.5Gl	10 g	10 mL	10 mL	0.5 mL	-	-
L@Gel/0.5Gl/PT	10 g	10 mL	10 mL	0.5 mL	0.5 mL (3 K U mL^−1^)	-
L@Gel/0.5Gl/PTxW	10 g	10 mL	10 mL	0.5 mL	0.5 mL (3 K U mL^−1^)	10 mL
L@Gel/2Gl	10 g	10 mL	10 mL	2 mL	-	-
L@Gel/2Gl/PT	10 g	10 mL	10 mL	2 mL	0.5 mL (3 K U mL^−1^)	-
L@Gel/2Gl/PTxW	10 g	10 mL	10 mL	2 mL	0.5 mL (3 K U mL^−1^)	10 mL

**Table 2:** Total weight loss for film samples over nine days, from the second day after casting.

Sample	Total % Weight Loss	Glycerol Content
L@Gel	1.26 ± 0.24 (a)	0%
L@Gel/0.5Gl	4.94 ± 0.20 (b)	5%
L@Gel/0.5Gl/PT	4.63 ± 0.14 (b)	5%
L@Gel/0.5Gl/PTxW	8.46 ± 0.39 (c)	5%
L@Gel/2Gl	2.73 ± 0.22 (d)	20%
L@Gel/2Gl/PT	1.86 ± 0.14 (a)	20%
L@Gel/2Gl/PTxW	5.44 ± 0.46 (e)	20%

Note. The results are presented as mean ± SD with groupings by Tukey’s HSD test; means sharing any letter are not significantly different.

**Table 3:** Film mechanical properties by durometer hardness test method, Shore A values.

Sample	Mean ± SD
L@Gel	83.3 ± 1.53 (c)
L@Gel/0.5Gl	75.0 ± 2.65 (b)
L@Gel/0.5Gl/PT	55.2 ± 2.75 (c)
L@Gel/0.5Gl/PTxW	52.1 ± 1.68 (c)
L@Gel/2Gl	50.0 ± 2.65 (c)
L@Gel/2Gl/PT	45.5 ± 3.0 (d)
L@Gel/2Gl/PTxW	41.0 ± 1.7 (d)

Note. The results are presented as mean ± SD with groupings by Tukey’s HSD test; means sharing any letter are not significantly different.

## Data Availability

The raw data supporting the conclusions of this article will be made available by the authors on request.

## Supplementary Materials

The following supporting information can be downloaded at: https://www.mdpi.com/article/10.3390/polym17162238/s1. Data gathered during experiments and measurements zipped and attached, contain: FTIR raw measurements files, NIR datasets, normalized and aggregated MOS sensor data, weight loss data, and hardness tests data in CSV format.
